# Correction: Heller et al. Characterization Methods along the Process Chain of Electrical Steel Sheet—From Best Practices to Advanced Characterization. *Materials* 2022, *15*, 32

**DOI:** 10.3390/ma16031196

**Published:** 2023-01-31

**Authors:** Martin Heller, Anett Stöcker, Rudolf Kawalla, Nora Leuning, Kay Hameyer, Xuefei Wei, Gerhard Hirt, Lucas Böhm, Wolfram Volk, Sandra Korte-Kerzel

**Affiliations:** 1 Institute of Physical Metallurgy and Materials Physics (IMM), RWTH Aachen University, 52074 Aachen, Germany; 2 Institute of Metal Forming (IMF), TU Bergakademie Freiberg, 09596 Freiberg, Germany; 3 Institute of Electrical Machines (IEM), RWTH Aachen University, 52052 Aachen, Germany; 4 Institute of Metal Forming (IBF), RWTH Aachen University, 52056 Aachen, Germany; 5 Chair of Metal Forming and Casting (utg), TU München, 85748 Garching, Germany

## Error in Figure

In the original publication [1], there was a mistake in Figure 16 as published. The corrected Figure 16 appears below.

## Text Correction

There was an error in the original publication [1]. In the course of further Recrystallization Scratch Experiments, we found a unit error in one of our Matlab scripts used to calculate the grain boundary mobility. At one point, the Vickers Hardness value (kgf/mm^2^) was not correctly transformed into SI units (1 kgf/mm^2^ = 9.80665 N/mm^2^). This affects one result of the paper [1] published with you. Please find attached a corrected version of Section 4.7, which is the only section affected. As you can see, the heat treatment parameters, the hardness value and the qualitative bar distribution have also changed slightly. This is because we had to start over with the raw data, with the old raw data no longer being fully available. Therefore, we switched to a closely related example. However, we believe that this does not significantly change the scope or message of the paper, as it is only exemplary data that can still be explained well. We apologise for the inconvenience and look forward to continuing our good working relationship. A correction has been made to **Section 4.7. Recrystallization Scratch Experiments:**

### 4.7. Recrystallization Scratch Experiments

Recrystallization during final annealing is one of the most important phenomena for electrical steel since the dislocation density is heavily reduced, the final texture develops, and the final grain size is adjusted. All three are very important factors for the final magnetic properties. Recrystallization is composed of nucleus formation and nucleus growth. Until now, how and where the nuclei form remains under discussion in the literature. It is, however, known that only nuclei of a critical size with mobile high-angle grain boundaries and a low dislocation density relative to the surrounding volume can grow. Such nuclei can form during deformation or recovery by the formation of substructures through the accumulation of dislocations. This means, conversely, that the nuclei do not form randomly or originate anew; they are already pre-existing before recrystallization and thus have orientations that evolve during rolling [27]. In order to be able to model recrystallization and grain growth on a physical basis, the nucleus formation process as well as the grain boundary mobility (grain growth) and the factors influencing both need to be understood. The following characterization methods, recrystallization scratch experiments and quasi-in situ EBSD (Sections 4.7 and 4.8) provide approaches to understanding these aspects better.

With the help of the first approach, the **grain boundary mobility** can be determined. Figure 15 shows a schematic layout of the experiment first proposed by Basu et al. [77].

First, a single crystal needs to be grown (Section 4.2). Next, a cuboid must be cut out of the single crystal, which is subsequently cold rolled to introduce a driving force (new dislocations) for recrystallization. For this, it is important that the single crystal’s orientation and the resulting ductility’s temperature dependence are taken into account because some orientations are so brittle that they fracture during rolling at room temperature before the driving force is high enough. A minimum degree of deformation of 80% is recommended. Below this value, recrystallization might not occur; however, this will of course strongly dependent on the material. Since there are no high-angle grain boundaries and possibly no shear or deformation bands in the single crystalline sheet, nucleation sites need to be introduced in addition to the cold rolling procedure. Bringing in a defined scratch on the surface after initial metallographic preparation results in nicely localised nuclei, which can be encouraged to grow during heat treatment. Good results for the scratch can be readily achieved in a well-equipped mechanical workshop; here, we simply dragged a hard metal lace fixed in a CNC machine across the sample at a depth of 0.06 mm and a speed of 13 m/min. Afterwards, the sample needs to be fully metallographically prepared for EBSD without grinding away the scratch. In order to derive the resulting driving force for recrystallization in the surrounding, cold-rolled volume, which is based on the dislocation density and is an important factor for the later mobility calculation, different techniques can come into play. There are indirect techniques, such as Vickers hardness (Section 3.2) or the measurement of geometrically necessary dislocation density by EBSD, as well as direct techniques to image dislocations, such as ECCI or TEM. For the former technique, Equations (2) and (3) from Section 3.2 must be combined and inserted into (7) in order to calculate the driving force (*F_dd_*-driving force based on the dislocation density (*ρ*)) [20]:

(7) $$F_{dd}=\alpha *G*b^{2}*\rho$$

After determining the driving force, the sample is ready for heat treatment. For this, it is important to use a reducing annealing atmosphere, e.g., forming gas, to prevent the prepared surface from oxidation, and the heat treatment parameters must be chosen carefully in order to trigger growth at the scratch but prevent nucleus formation in the matrix. Results from one successful recrystallization scratch experiment can be found in Figure 16. In the upper third of (a) the single-coloured matrix (purple) can be seen containing some substructures (dark lines); in the lower third, the non-indexed scratch (black) is visible and, in the middle, nuclei grow from the scratch into the matrix. Through combining the free growth length of the nuclei *r*_gl_, the nuclei orientation, the heat treatment parameters (temperature 765 °C and time *t =* 300 s) and the driving force *F_dd_* (calculated based on HV_0.2/15_ = 303.9), the mobilities of grain boundaries, classified according to their misorientation angle *g,* can be calculated (Figure 16b). Grain boundary misorientations are only measured between nuclei and the matrix.

These first results of an Fe2.4wt.%Si sample (Table 2) are about two orders of magnitude below the results of the investigations of Wits et al. [78], who studied the body-centred cubic/face-centred cubic interface mobility in iron, and of Kim et al. [79] who used the aforementioned results for the grain boundary mobility during texture development investigations in body-centred cubic iron sheets. The reasons for the lower measured mobility could be surface effects, a general influence of the alloying elements (Si, Al, Mn etc.), solute-drag (C, N etc.), an overestimation of the driving force based on Vickers Hardness (~26 MPa), or the mobility is overestimated in the few studies available for this material. However, the qualitative shape of the distribution in Figure 16b, with very low mobilities for low angle grain boundaries (0–15°), a high point between 30–50° and a steep descent towards 90° is typical for cubic metals [20,80]. Future analyses could focus more on the role of the rotation axis or special grain boundaries in body-centred cubic materials. The parameters, which can and should be varied in this kind of experiment to learn more about the influencing factors, are: the alloy concept (and thereby the extent of segregation to grain boundaries and solute drag [81]), the degree of deformation and heat treatment parameters. In this way, the method provides a viable route to collect grain boundary mobility data for many boundaries at the same time and allows an efficient comparison of different alloys or processing conditions, as is urgently needed for the physical modelling of microstructure evolution during the heat treatment of rolled, polycrystalline electrical sheets.

The authors apologize for any inconvenience caused and state that the scientific conclusions are unaffected. This correction was approved by the Academic Editor. The original publication has also been updated.

## Figures and Tables

**Figure 16:**
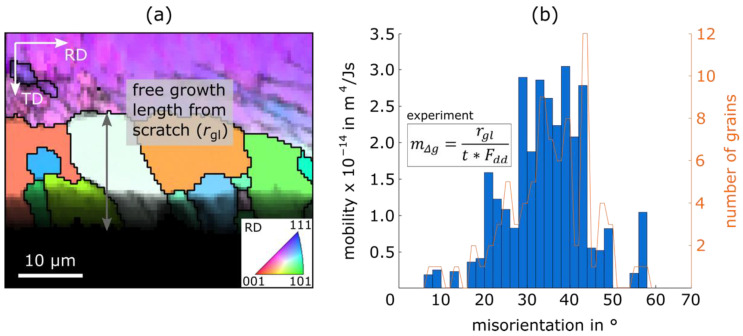
(**a**) Exemplary section from an EBSD panorama image heat-treated at 765 °C for *t* = 300 s, with one free growth length *r*_gl_ from the scratch drawn in, coloured according to an IPF || RD and (**b**) results of experimental grain boundary mobility calculations (plus equation) based on over 80 grains divided into misorientation classes Δ*g*, with a width of 2°. IPF—inverse pole figure.
